# Efficiency over thoroughness in laboratory testing decision making in primary care: findings from a realist review

**DOI:** 10.3399/bjgpopen20X101146

**Published:** 2021-02-03

**Authors:** Claire Duddy, Geoff Wong

**Affiliations:** 1 Nuffield Department of Primary Care Health Sciences, University of Oxford, Oxford, UK

**Keywords:** realist review, realist synthesis, clinical laboratory techniques, primary health care, practice patterns, physicians, clinical decision making, general practice

## Abstract

**Background:**

Existing research demonstrates significant variation in test-ordering practice, and growth in the use of laboratory tests in primary care. Reviews of interventions designed to change test-ordering practice report heterogeneity in design and effectiveness. Improving understanding of clinicians’ decision making in relation to laboratory testing is an important means of understanding practice patterns and developing theory-informed interventions.

**Aim:**

To develop explanations for the underlying causes of patterns of variation and increasing use of laboratory tests in primary care, and make recommendations for future research and intervention design.

**Design & setting:**

Realist review of secondary data from primary care.

**Method:**

Diverse evidence, including data from qualitative and quantitative studies, was gathered via systematic and iterative searching processes. Data were synthesised according to realist principles to develop explanations accounting for clinicians’ decision making in relation to laboratory tests.

**Results:**

A total of 145 documents contributed data to the synthesis. Laboratory test ordering can fulfil many roles in primary care. Decisions about tests are incorporated into practice heuristics and tests are deployed as a tool to manage patient interactions. Ordering tests may be easier than not ordering tests in existing systems. Alongside high workloads and limited time to devote to decision making, there is a common perception that laboratory tests are relatively inconsequential interventions. Clinicians prioritise efficiency over thoroughness in decision making about laboratory tests.

**Conclusion:**

Interventions to change test-ordering practice can be understood as aiming to preserve efficiency or encourage thoroughness in decision making. Intervention designs and evaluations should consider how testing decisions are made in real-world clinical practice.

## How this fits in

Research on laboratory testing has long demonstrated variation and growth in the use of tests. Existing reviews have identified lists of factors associated with test-ordering behaviour, and mixed evidence of the effectiveness of interventions designed to change testing practice. This realist review presents explanations for clinicians’ test-ordering behaviour, illustrating the wide range of influences affecting decision making about laboratory testing, and highlighting the combined effect of high workload and a generalised perception that laboratory tests are relatively trivial and inconsequential interventions. As a result, clinicians often prioritise efficiency and pragmatism over thoroughness in making decisions about the use of laboratory tests, focusing their limited time and resources elsewhere. Future intervention designs and evaluations should take account of real-world practice by recognising that making changes to wider systems may not change perceptions of laboratory testing, and that encouraging thoroughness in decision making in this area of practice may have unintended consequences elsewhere.

## Introduction

Existing research has long demonstrated growth in the use of laboratory tests in primary care and the existence of variation in test-ordering practice.^1–6^ These patterns raise important questions about how much variation in clinical decision making is warranted, and whether increased testing improves health outcomes and represents cost-effective use of scarce resources. In the UK, NHS Improvement estimates expenditure of £2.2 billion annually on pathology services.^7^ Primary care makes a significant contribution: in 2006, the Carter Review estimated that 35%–45% of requests for laboratory tests originated in primary care;^8^ and in 2014, NHS England estimated that over 50 million electronic reports of laboratory test results were delivered to GPs each year.^9^

Laboratory testing often represents an early step in a clinical pathway, carrying further consequences for downstream activity.^10^ Both undertesting and overtesting can result in negative consequences for patients. Undertesting can mean delayed or missed diagnoses, and a lack of monitoring of long-term conditions or medication side effects. However, growth in test use raises concerns about overtesting: some testing may be unnecessary because of the low likelihood of benefitting patients,^11^ or may even cause harm; for example, unnecessary testing has the potential to increase patient anxiety, raises the chances of false positive results, and has the potential to provoke ‘cascades’ of further unnecessary investigations and interventions.^10,12,13^

Multiple reviews have attempted to assess the effectiveness of a range of interventions designed to change test-ordering behaviour.^14–23^ These reviews report heterogeneity in intervention designs, effectiveness, and the sustainability of changes in practice. Across the reviews, the most frequently measured outcomes relate to test-ordering activity and behaviour. There is an emphasis on reducing test ordering or improving 'appropriateness'. The latter often refers to assessments of whether or not test-ordering activity fits within existing guidelines (see Table 1 below for more detail). Observed variation in practice and in the impact of interventions suggests multiple causal mechanisms may underlie test-ordering decisions, and that context may play an important role in determining outcomes. Understanding the causes of observed patterns of testing is an important means of informing the development and evaluation of interventions that aim to change practice. The realist approach adopted here aimed to: produce explanations that account for observed patterns and the influence of context; identify the underlying causal mechanisms that underlie test-ordering practice; and produce recommendations to guide future research.^24^

**Table 1.** Summary of interventions and outcomes assessed in studies included in existing systematic reviews

		**Interventions prioritising** **efficiency**	**Interventions prioritising thoroughness**
**Review**	**Test-ordering outcome(s**)	**Process changes (including computer systems**)	**Guidelines and/or protocols**	**Education**	**Audit and feedback**	**Financial incentives**
Solomon *et al* 1998^14^	Reduction in test-ordering volumeReduction in test expenditure	x	x	x	x	x
Main *et al* 2010^15^	Changes in test-ordering volume‘Appropriateness’ of testing	x				
Smellie 2012^16^	Reduction in test-ordering volumeReduction in test expenditure‘Appropriateness’ of testing	x	x	x	x	x
Cadogan *et al* 2015^17^	Reduction in test-ordering volume	x	x	x	x	
Kobewka *et al* 2015^18^	Reduction in test-ordering volume	x		x	x	x
Thomas *et al* 2015^19^	Reduction in test-ordering volume	x	x		x	
Thomas *et al* 2016^20^	Change in test-ordering volume	x		x	x	
Zhelev *et al* 2016^21^	Reduction in test-ordering volumeChanges in test expenditure‘Appropriateness’ of testingChanges in testing patterns	x	x	x	x	x
Delvaux *et al* 2017^22^	‘Appropriateness’ of testingChanges in test expenditureClinical outcomes	x				
Maillet *et al* 2018^23^	Changes in test ordering‘Appropriateness’ of testingWorkload	x				

## Method

A realist review is an interpretive form of evidence synthesis, conducted with the aim of identifying and synthesising relevant and trustworthy data that can be used to develop a better understanding of its subject. Realist analysis can be used to develop explanatory theory (called ‘programme theory’), which takes account of important influences of context and identifies underlying causal mechanisms that produce observed outcomes.^24^ This realist review aimed to develop explanations for primary care clinicians’ decision making about laboratory tests, and to make theory-informed recommendations for future research and intervention design.

The methods for this review are described in detail in the published protocol.^25^ The review was conducted according to Pawson’s five steps,^26^ outlined briefly in Table 2 below and in detail in supplementary **Table S1**. Review processes adhered to the RAMESES quality^27^ and reporting^28^ standards throughout.

**Table 2.** Summary of realist review methodology

**Step 1**Initial programme theory (IPT) development	An IPT is a first attempt to develop an understanding of the research question. To develop the IPT for this review, two scoping searches were run of the literature to identify: (a) existing theoretical perspectives; and (b) common intervention designs in relation to test-ordering practice. Full details of the search strategies are provided in supplementary **Table S2**. The IPT was further developed via the input of the stakeholder group and is presented in full in supplementary **Figure S1**.
**Step 2**Searching for evidence	The main search for evidence was undertaken with the aim of assembling a body of relevant data that could be used to develop and refine the programme theory. A broad range of sources were searched (*n* = 15) to ensure that literature across multiple disciplines was considered. Full details of the main search strategy are provided in supplementary **Table S3**.Additional documents were identified via supplementary search methods such as citation tracking (snowballing) and via personal contacts and networks.^69,70^Further searches were undertaken later to identify relevant substantive theory to act as a theoretical lens through which to understand the review’s overall findings.^71^ The search strategies employed are provided in supplementary **Table S4**.
**Step 3**Selection and appraisal	Document selection was based on an assessment of relevance (whether or not documents contained data that could be used to develop theoretical explanations [‘programme theory’]) and rigour (whether data were considered credible in relation to their role in contributing to the theory).^26,72^Included documents provided data on important contexts, mechanisms, and outcomes related to clinician decision making in relation to laboratory test ordering in primary care settings, or provided data related to analogous settings or decisions, or relevant theoretical perspectives. More details on data selection processes are provided in supplementary **Table S1**.
**Step 4**Data extraction and organisation	Included documents were read closely and coded in NVivo (version 12 Pro) to organise the data and identify important concepts that could inform the realist analysis. The characteristics of included documents (*n* = 145) are provided in supplementary **Table S5**.
**Step 5**Analysis and synthesis	Analysis and synthesis of included data involved the iterative development of realist ‘context–mechanism–outcome configurations’ (CMOCs).These are theoretical causal explanations describing how important contexts trigger the mechanisms that generate observed outcomes. Members of the stakeholder group provided feedback on the relevance and resonance of the developing theories. CMOC development and refinement continued until the reviewers agreed theoretical saturation was reached.A ‘final programme theory’ (FPT) was developed after consideration of the full set of CMOCs and drawing on theoretical literature.Full details of the CMOCs developed and illustrative data excerpts are provided in supplementary **Tables S6–S8**.

A group of 14 stakeholders, including patients, members of the public, clinicians, a laboratory scientist, and a policymaker were involved from the outset. Their direct knowledge and experience of test-ordering practice shaped the review, contributing to the development of the initial and refined programme theories.

## Results

**Figure 1.**
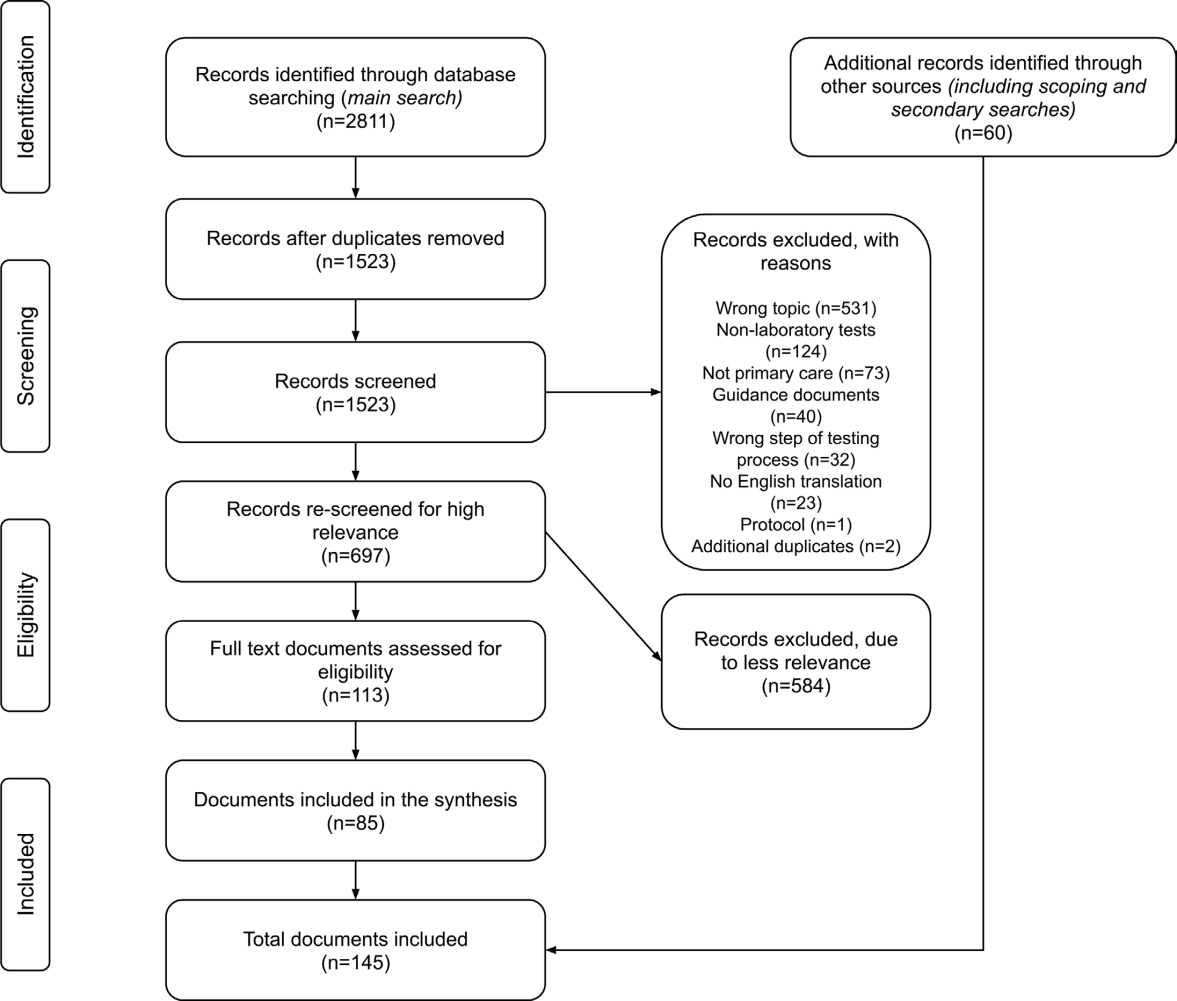
Document screening and selection processes

A total of 145 documents contributed data to this review; see Figure 1. Most reported research studies (*n* = 123), but grey literature including commentaries (*n* = 20) and theoretical work (*n* = 2) were also included. Documents describing research comprised 60 cross-sectional or survey studies, 27 qualitative studies, 12 narrative reviews, nine systematic reviews, six cohort studies, five decision-analysis studies, two randomised controlled trials, one project evaluation, and one case report. Full details of the included documents are provided in supplementary **Table S5**.

The final programme theory developed from the realist analysis is presented below. This explanatory framework is underpinned by three overarching context–mechanism–outcome configurations (CMOCs) developed during the review, summarised in Table 3. The three overarching CMOCs were developed from the detailed analysis of 52 underpinning CMOCs in all (presented in full in **Tables S6–S8**).

**Table 3.** Summary of realist analysis

**Overarching CMOCs**	**Illustrative examples of underpinning CMOCs**
When laboratory tests are perceived to be relatively trivial (C1), and cognitive resources are limited (C2), clinicians prioritise efficiency over thoroughness for test-ordering decisions, directing their cognitive resources to other clinical decisions (M) so **decisions about testing will be based on heuristics or routines** (O).	When clinicians have incomplete technical knowledge about laboratory medicine and/or diagnostic reasoning (C), they rely on ‘gist’ understanding (M) to develop decision-making heuristics for test ordering (O) [CMOC 1b].In the presence of diagnostic uncertainty (C), clinicians may apply a heuristic of 'more testing is better' (O1) or 'rule out the worst case' (O2) as they seek to minimise the risk of missing a diagnosis (M) [CMOCs 2a–2b].When a test or condition is 'in fashion', and there is high awareness among clinicians and/or the public (C), the use of this test may be incorporated into testing heuristics (O) owing to increased awareness (‘salience’) (M) [CMOC 3g].
When laboratory tests are perceived to be relatively trivial (C1), and cognitive resources are limited (C2), clinicians prioritise efficiency over thoroughness for test-ordering decisions, and direct their cognitive resources to other clinical decisions (M) and so **tests may be used to fulfil social and strategic functions** (O).	In the presence of diagnostic uncertainty (C), clinicians may demonstrate care (M1), attempt to reassure (M2), or exert control via ‘doing something’ for their patients (M3) by ordering tests (O) [CMOCs 5a–5c].When clinicians anticipate a 'difficult' interaction with a patient (C), they may use the offer of a laboratory test (O) as a strategy to help manage the consultation (M) [CMOC 6c].When clinicians anticipate disagreement with a patient about their proposed management plan (C), they may acquiesce to patient requests or expectations and order tests (O) to avoid having to explain why they are inappropriate (M1) or avoid conflict in the consultation (M2) [CMOCs 7b–7c].
When laboratory tests are perceived to be relatively trivial (C1), and cognitive resources are limited (C2), clinicians will prioritise efficiency over thoroughness in test-ordering decisions, and direct their cognitive resources to other clinical decisions (M) so **decisions about testing will be open to wider system influences** (O).	When responsibility for patient care is shifted from secondary to primary care (C), clinicians in primary care settings comply with testing expectations and requests (M) received from secondary care and take on responsibility for associated testing (O) [CMOC 9c].In the absence of disincentives for inappropriate testing (C), clinicians and laboratory managers will not prioritise concerns about under/overtesting (M) and so will not take action to address these problems (O) [CMOC 10b].When tests are available to order as part of profiles or panels (C), clinicians may try to save time and cognitive energy (M) by ordering full panels instead of individual tests (O) [CMOC 11b].

C = context; M = mechanism; O = outcome

### Competing demands, relative triviality, and decision making

The data included in this review demonstrate that laboratory test ordering fulfils a wide range of roles for clinicians. Test-ordering decisions may be built into clinicians’ practice heuristics and fulfil numerous social or strategic roles in managing patient interactions. It is also clear that features of the wider environment — including computer systems, organisational structures, and social or cultural norms — often tend to encourage (or fail to discourage) the use of laboratory tests.

Underpinning these findings are two important overarching contexts: clinicians are juggling heavy workloads and limited time with patients; and laboratory tests are often considered to be relatively trivial and inconsequential interventions.^29–35^ Some data even suggest that where there are obvious negative consequences of testing, these may be construed positively by clinicians and patients.^36,37^

Busy clinicians with limited time and attention to devote to many competing tasks must prioritise. Hollnagel’s Efficiency–Thoroughness Trade-Off (ETTO) principle provides a framework for understanding this. For example:

*'* *In their daily activities* *…* *people (and organisations) routinely make a choice between being efficient and being thorough, since it is rarely possible to be both at the same time.* *'* ^38^

**Figure 2.**
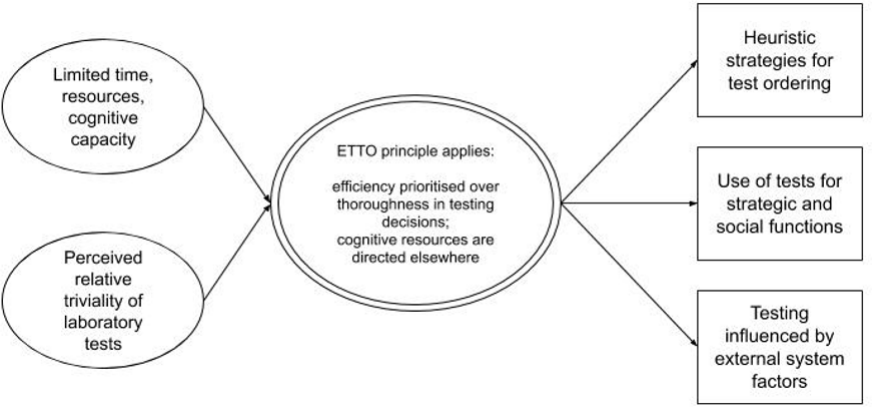
Final programme theory illustrating overarching context–mechanism–outcome configurations (CMOCs). ETTO = Efficiency–Thoroughness Trade-Off. Figure legend: single line oval = context, double line oval = mechanism, rectangle = outcome.

On the ETTO spectrum, ‘thoroughness’ is understood to confer safety, while increases in ‘efficiency’ sacrifice diligence in favour of saving time and psychological effort. When laboratory tests are considered relatively trivial or inconsequential, clinicians trading off between efficiency and thoroughness may not believe there will be any significant loss of safety in relation to increasing efficiency in decisions about testing. Taking this position permits the application of efficient heuristics and use of tests for social or strategic purposes, and means that clinicians are unlikely to expend effort in working against wider pro-testing systems; see Figure 2.

Some data pointed to exceptions: clinicians do resist pressures to test, when they perceive that tests may carry burdens or harms for patients, or when they have adopted professional identities or follow norms associated with more conservative or parsimonious practice (see CMOCs 8a–c, 10c in supplementary **Tables S6–S8**). In such cases, laboratory tests are no longer considered trivial, but are understood to carry real, potentially harmful consequences. However, in these circumstances, clinicians face the same pressures of high workloads and limited time. From the included data, it is unclear if the outcome is more thorough decision making about test ordering (and potentially the prioritisation of efficiency in other areas to compensate) or simply the adoption of heuristics that favour not testing.

In the case of laboratory testing in primary care settings, the dominant mechanism of prioritising efficiency over thoroughness tends to lead to sustained and increasing use of tests.

## Discussion

### Summary

This realist review demonstrates the complexity of laboratory test-ordering practice in primary care. Clinicians use tests to fulfil a variety of roles. Their test-ordering decisions are affected by a wide range of contextual factors and generated by many different motivations. Overall, a commonly held perception of laboratory tests as relatively trivial and inconsequential interventions often permits clinicians to prioritise efficiency over thoroughness in test-ordering decisions.

The diversity of roles that laboratory testing can fulfil helps to explain variation in test use as the result of variation in multiple contextual circumstances. Variation can also be understood as resulting from a widespread context of perceived relative triviality, which acts to enable this diversity of motivations for testing and variation in individuals’ decision making. However, it is also clear that the data suggest that the prioritisation of efficiency in test-ordering practice tends to lead to increased and self-sustaining use of tests. Increasing test use should be seen within the context of shifting norms and expectations in clinical practice, and broader cultural beliefs in the benefits and capabilities of testing and health care.^39^

It should also be understood in the context of primary care workloads,^40,41^ and the need for clinicians to find efficient ways of practising with limited resources.^42–44^ The review’s final programme theory offers a novel framework to understand common patterns of test-ordering behaviour: clinicians with limited time and energy who consider laboratory tests to be relatively trivial interventions are likely to prioritise efficiency over thoroughness in these decisions. They are likely to devote more time and psychological effort to other areas of practice, and are unlikely to expend resources in resisting the multiple societal and system features that tend to incentivise testing.

### Strengths and limitations

This review included a wide range of evidence obtained via systematic searching. The analysis was enhanced by ‘borrowing’ relevant data from documents focused on other areas of clinical practice and drawing on substantive theory to generate new insight. The involvement of a diverse group of stakeholders helped to shape the project and refine the analysis, ensuring its relevance and resonance for real-world practice and policy.

As in all reviews, findings were limited by the available data. Some plausible explanations for test-ordering behaviour proposed by stakeholders remain unsubstantiated; for example, the question of whether decision making is affected by clinicians’ emotional affect, and whether defensive mechanisms may lead to decreased testing where clinicians reason it is best to avoid opening Pandora’s box. The available data did not permit determination of which CMOCs are more dominant or explanatory than others. A representative set of CMOCs has been generated offering explanations for observed patterns, but future research may lead to the refinement, confirmation, or refutation of these explanatory theories.

The included literature was variable in quality, and the data underpinning each individual CMOC varies in volume and type. The full details of the contributing data are provided in supplementary **Tables S5–S8** to permit the reader to make judgements about the strength of the evidence underpinning the analysis.

### Comparison with existing literature

Two earlier reviews have collated studies of factors affecting test ordering, similarly highlighting the wide range of influences affecting decision making in this area of practice, including clinicians’ experience and attitude toward risk.^45,46^ The detailed realist analysis in this review concurs with this image of complexity in test-ordering decision making and extends existing work by focusing on the role of important contexts and the multiple mechanisms that generate clinicians’ test-ordering behaviour. The CMOCs presented in supplementary **Tables S6–S8** provide a set of testable theories about clinicians’ test-ordering practice that may be refined, confirmed, or refuted by further research. In addition, the final programme theory presented above provides an overarching framework through which to understand the complex picture of testing practice. The application of the ETTO principle as a theoretical lens permits common patterns of variation in test use and growth in the use of tests to be better understood.

The review’s findings also have parallels in research conducted in other areas of practice. The ETTO principle has been usefully applied in qualitative studies in primary care, to understand the conduct of medication reviews,^44^ and the management of test results,^47^ and prescription requests.^43^ There are also similarities with findings from other studies of clinical decision making, especially those that employ dual-processing theory to understand how decisions are made.^48,49^ The addition of this review’s findings further validates the utility of the ETTO principle as a means of understanding the realities of decision making and the prioritisation of workloads in primary care.

### Implications for research

The overall complexity of laboratory test-ordering practice and the perspective provided by the ETTO principle carry important implications for research, and especially for future intervention design and evaluation. The ETTO framework provides a novel way of categorising families of interventions that aim to influence clinical practice, as aiming either to preserve or increase efficiency, or to encourage thoroughness in decision making. To illustrate this point, the common intervention designs described in the studies included in existing systematic reviews of interventions designed to change test-ordering behaviour are summarised in Table 1.

#### Efficiency

Intervention designs involving making changes to test-ordering systems (especially order forms) or providing decision support at the point of ordering fall into this category. They aim to adjust clinicians’ heuristics or routines by reconfiguring decision-making options, making ‘appropriate’ testing easy and efficient, or ‘inappropriate’ testing more difficult. Underlying such interventions is the often implicit theory that clinicians do prioritise efficiency, that is, they base test-ordering decisions on heuristics, informed by their own experience, practice norms, and system constraints, in a similar manner to observed behaviour in relation to guideline adherence.^50^

Reviews report variable effectiveness for interventions that aim to preserve or increase efficiency in primary care settings, although many are reported to result in reductions in testing volumes.^14–21^ One study reports that where tests were added to order forms, usage increased,^51^ and several studies have demonstrated that adding reminders or alerts can change testing behaviour,^52–59^ although 'alert fatigue' may be a problem.^22,60–63^

The wider consequences of relying on decision-making heuristics, and of attempts to impose changes on these routines, which may be ‘good enough’ and help primary care clinicians manage competing demands and challenging conditions, are unclear and deserve attention. Decision-making heuristics may permit both sound and efficient decision making,^64^ or may be vulnerable to errors resulting from cognitive biases.^65^ They may also have unintended and unforeseen consequences affecting, for example, clinicians’ workloads and relationships with patients over the longer term. Future research, including ethnographic studies of practice and long-term observational studies, could be used to describe in detail, and ultimately assess the reliability of clinicians’ testing heuristics, but should prioritise the need to assess patient outcomes associated with testing, rather than the common surrogates of testing volumes or adherence to guidelines (which are frequently consensus based).^66,67^

#### Thoroughness

Other interventions seek to focus clinicians’ attention on test-ordering practice by adding processes, delivering education, introducing financial incentives, or providing feedback on testing behaviour. As above, reviews report variable effectiveness, although many interventions are successful in changing testing behaviour to some degree.^14–21^ As a group, these interventions represent attempts to provoke thoroughness, by providing additional information to factor into decisions or engineering opportunities for reflection on practice. The findings of this review suggest that clinicians’ responses may be affected by individual efficiency–thoroughness trade-offs (which may differ from normal practice under trial conditions), informed by perceptions of the relative triviality of laboratory tests, and constrained by workload and competing demands. Where interventions can change one of these important contexts — in practice, perceptions of triviality may be more amenable to change than prevailing conditions of high workload and limited resources — they may be more likely to be effective. Different strategies may produce effects via different mechanisms. Several reviews have reported that multi-component interventions are the most effective in changing test-ordering behaviour.^14,16–18^ Complex interventions may act to exert greater pressure to change perceptions of laboratory testing, provoking multiple mechanisms that ‘work’ for different individuals and reflecting the wide variation in factors that influence clinicians’ testing practice.

Where interventions are unsuccessful, the authors' programme theory suggests that this may reflect a failure to convince clinicians that laboratory testing is important, and/or that the time and resources for thorough decision making were not available.^20,21^ It may, therefore, be instructive for future evaluations of interventions to attempt to uncover which mechanisms and associated intervention strategies have been effective in which contexts, and to include an assessment of outcomes relating to clinicians’ perceptions of the relative triviality or importance of laboratory testing and workloads.

Finally, the complexity of test-ordering practice requires that future research should consider the potential unintended consequences of interventions designed to change test-ordering practice. For example, where intervention design aims to reduce test ordering for social or strategic purposes, evaluations should ensure that potential trade-offs in efficiency and thoroughness are considered; for example, what are the side effects (in relation to clinicians’ workloads, as well as for patients) of preventing or encouraging clinicians to avoid deploying tests in this way? Approaches that permit theory-informed intervention design and evaluations that take account of complexity, differences in context and unintended consequences are recommended, especially realist evaluation.^68^
